# Genome-wide association study and genomic prediction of leaf spot (*Stemphylium vesicarium*) resistance in spinach diversity panel

**DOI:** 10.3389/fpls.2025.1663650

**Published:** 2025-09-03

**Authors:** Gehendra Bhattarai, Bo Liu, James Correll, Ainong Shi

**Affiliations:** ^1^ Department of Horticulture, University of Arkansas, Fayetteville, AR, United States; ^2^ Department of Entomology and Plant Pathology, University of Arkansas, Fayetteville, AR, United States; ^3^ University of New Hampshire, Cooperative Extension Food and Agriculture, Kendall Hall, Durham, NH, United States

**Keywords:** disease resistance, genome-wide association study, spinach, semphylium leaf spot, genomic prediction (GP)

## Abstract

Stemphylium leaf spot (SLP), caused by *Stemphylium vesicarium*, has emerged as an increasing threat to spinach production in the United States, with widespread outbreaks reported across major spinach-growing regions over the past two decades. The objectives of this study were to: (1) evaluate global USDA spinach germplasm collections and commercial cultivars for resistance to *S. vesicarium*; (2) perform genome-wide association studies (GWAS) to identify genomic regions associated with resistance; and (3) conduct genomic prediction (GP) to enhance selection accuracy. A total of 311 diverse spinach genotypes, including USDA germplasm accessions and commercial cultivars, were evaluated under greenhouse conditions at the University of Arkansas using the *S. vesicarium* isolate Sb-1-St001 from 2019 to 2021. The panel exhibited a wide range of disease responses. GWAS using disease severity index (DSI) values and whole-genome resequencing (WGR)-based SNP markers identified four SNPs—SOVchr1_127757911 (127,757,911 bp, Chr1), SOVchr2_21962694 (21,962,694 bp, Chr2), SOVchr4_114674293 (114,674,293 bp, Chr4), and SOVchr5_37417509 (37,417,509 bp, Chr5)—that were significantly associated with DSI for SLP resistance. Genomic prediction of DSI was performed using seven GP models across nine randomly selected SNP datasets and two GWAS-derived SNP sets. The GWAS-derived marker sets produced higher prediction accuracies in cross-population prediction, with r-values of 0.45 and 0.51 for the 4- and 18-SNP sets, respectively. These results underscore the potential of marker-assisted selection (MAS) and genomic selection (GS) to accelerate the development of spinach cultivars resistant to Stemphylium leaf spot.

## 1 Introduction

Spinach (*Spinacia oleracea* L.) is an important leafy vegetable crop. Due to its nutritional benefits and the availability of fresh and frozen clean, bagged products, spinach consumption has steadily increased over recent decades.

Stemphylium species cause leaf spot diseases and infect a wide range of hosts, including tomato (Su et al., 2019), lentils (Saha et al., 2010; Podder et al., 2013), cucumber (Vakalounakis and Markakis, 2013), onion (Dangi et al., 2019), parsley (Koike et al., 2013), and spinach (Correll et al., 1994; Koike et al., 2001). While leaf spot diseases primarily reduce yield through foliar damage in many crops, the impact is more severe in leafy vegetables like spinach, where such symptoms render the product unmarketable. In spinach, the disease is highly host-specific and significantly reduces both quality and yield, especially for the fresh market, posing a major constraint to production (Correll et al., 1994).

Stemphylium leaf spot (SLP) in spinach was first reported in the Salinas Valley, California, in 1997 (Koike et al., 2001), and subsequently in other states, including Washington and Oregon (du Toit and Derie, 2001), Florida (Raid, 2001), Maryland and Delaware (Everts and Armentrout, 2001), Arizona (Koike et al., 2005), and Texas (Reed et al., 2010). Initial symptoms appear as circular, gray-green spots that later turn light tan with a papery texture and tend to coalesce. Sporulation is generally absent (Koike et al., 2001). *Stemphylium* leaf spot has become a major foliar disease in U.S. spinach production areas, including Arizona, California, South Carolina, and Texas (Liu et al., 2020a, b). The causal organism was originally identified as *Stemphylium botryosum* Wallr., and later designated *S. botryosum* f. sp. *spinacia* due to its host specificity (Koike et al., 2001, 2005). More recently, two other species—*S. vesicarium* and *S. beticola*—have also been reported as spinach pathogens, distinguishable by symptom characteristics, conidial morphology, DNA sequences, and sometimes the presence of a brown ring within lesions (Liu et al., 2020b). In recent years, SLP has become a serious issue in baby leaf spinach production in several states, including Arizona, California, South Carolina, Texas, and Florida (Wadlington et al., 2018; Liu et al., 2020b), especially under humid conditions. The increasing demand for fresh-market spinach over the past two decades has driven high-density planting, typically at 5–10 million seeds per hectare in key production regions (Bhattarai et al., 2020a; Dhillon et al., 2020). Such practices promote dense canopy formation, prolonged leaf wetness, poor air circulation, and high humidity—conditions favorable for foliar diseases, including SLP (Koike et al., 2001; Hernandez-Perez and du Toit, 2006; Wadlington et al., 2018; Liu et al., 2020b). As spinach acreage and production for fresh markets have significantly increased in the U.S. over the past three decades, quality standards have also tightened, with zero tolerance for leaf spot symptoms on baby leaf spinach, making diseased leaves unmarketable (Morelock et al., 2005).

Initial screenings of USDA spinach germplasm and commercial cultivars found no genotype completely immune to *SLP* caused by *S. botryosum* (Mou et al., 2008). However, some cultivars and accessions with higher levels of tolerance to local isolates were identified in Florida (Wadlington et al., 2018). Eight SNP markers associated with resistance were previously reported (Shi et al., 2016). Genetic resistance remains the most sustainable strategy for disease management, particularly for organic and conventional spinach production systems.

Genome-wide association studies (GWAS) have become a powerful tool for identifying genetic variants associated with target traits in natural and segregating populations. In spinach, GWAS has been successfully applied to map resistance loci for downy mildew (Bhattarai et al., 2020b, 2021; Cai et al., 2021), white rust (Shi et al., 2022), and *Stemphylium* (Shi et al., 2016). Additionally, resistance mapping in other crops, including tomato, has identified major-effect genes conferring resistance to *Stemphylium lycopersici*. For example, silencing an NBS-LRR gene eliminated resistance to gray leaf spot in tomato (Yang et al., 2022), further underscoring the importance of molecular markers in disease resistance breeding (Saha et al., 2010; Su et al., 2019).

Genomic selection (GS) is an emerging strategy that uses genome-wide markers to predict the breeding value of individuals, enabling selection without phenotyping or field trials (Meuwissen et al., 2001; Heffner et al., 2009; Bernardo, 2010; Jannink et al., 2010). GS has been applied to both qualitative and quantitative traits in various crop species, including horticultural and agronomic crops, using biparental, multiparent, and natural populations (Lorenzana and Bernardo, 2009; Heffner et al., 2011; Gezan et al., 2017; Poudel et al., 2019; Islam et al., 2020; Sehgal et al., 2020). Recently, GS was explored in spinach for white rust resistance (Shi et al., 2022). Several parametric models—such as ridge regression BLUP (rrBLUP), Bayes A, Bayes B, and Bayesian LASSO—and non-parametric models like Random Forest (RF) and Reproducing Kernel Hilbert Space (RKHS) are used to improve prediction accuracy. These models differ in assumptions regarding marker effects and trait inheritance, and their performance varies depending on the number and effect size of QTLs. For example, some models perform better for traits controlled by a few large-effect loci, while others are suited for complex traits governed by many small-effect alleles. Prediction accuracy is also influenced by factors such as trait heritability, population size, relatedness between training and testing sets, marker density, and linkage disequilibrium (Lorenzana and Bernardo, 2009; Asoro et al., 2011; Daetwyler et al., 2013; Habier et al., 2013; Poland and Rutkoski, 2016).

Given the increasing economic losses caused by SLP and the lack of complete resistance in commercial cultivars, there is a critical need to enhance our understanding of the genetic basis of resistance to *Stemphylium vesicarium* in spinach. Traditional screening approaches have been useful in identifying tolerant lines, but they are limited in scalability and often confounded by environmental variation. The integration of genomic tools such as GWAS and genomic selection (GS) offers a promising path to accelerate resistance breeding by identifying key loci and predicting resistant genotypes with high accuracy. However, the genetic architecture of SLP resistance remains poorly characterized, and there is limited information on the effectiveness of GS for this trait in spinach. A comprehensive study that combines GWAS with GS is therefore essential to develop robust, marker-informed strategies for breeding spinach cultivars with durable resistance to SLP. The objectives of this study were to: (1) evaluate a global collection of spinach germplasm accessions and commercial cultivars for resistance to *SLP* under greenhouse conditions; (2) identify genomic regions associated with resistance using GWAS; and (3) optimize genomic selection models for accurate prediction of resistance. This study provides new molecular resources, predictive models, and marker sets to support the development of *Stemphylium*-resistant spinach cultivars.

## 2 Materials and methods

### 2.1 Plant material

A total of 311 spinach accessions were evaluated for resistance to *Stemphylium vesicarium*, the causal agent of SLP, under greenhouse conditions at the Harry R. Rosen Alternative Pest Control Center (ROSE) on the University of Arkasnas Campus from 2019 to 2021. This collection included 271 USDA spinach germplasm accessions, 35 commercial cultivars, and five breeding lines developed at the University of Arkansas. The USDA germplasm accessions were originally collected from 32 countries, with more than ten accessions each from Turkey, the United States, Afghanistan, China, Macedonia, India, and Belgium (**Supplementary Tables S1a, b**; hereafter, ‘S’ denotes both supplementary tables and figures in the text). Ten seeds of each accession were sown in pots (10 cm diameter × 10 cm height) filled with LC1 potting mix (Sungro Horticulture Distribution Inc., Agawam, MA). After germination, plants were thinned to three per pot. Each treatment was replicated three times, with three pots per accession evaluated in each trial.

### 2.2 Inoculation and phenotyping

Spinach plants were inoculated with the *S. vesicarium* isolate Sb-1-St001 following the protocol described by (Liu et al. 2020a, b). Briefly, the isolate was cultured on potato dextrose agar (PDA) plates for 14 to 15 days. Conidia were harvested by washing the colony surface with distilled water, filtering the suspension through two layers of cheesecloth, and adjusting the spore concentration to 1 × 10^5^ spores/mL. A 0.01% Tween-20 solution was added to both the conidial suspension and the distilled water used for control treatments.

Thirty-day-old spinach plants were sprayed with the spore suspension using a Badger Basic Spray Gun (Model 250), applying a total volume of 50 mL per tray containing 18 pots. Non-inoculated control plants (cv. Viroflay) were sprayed with distilled water containing 0.01% Tween-20 and subjected to identical environmental conditions. After inoculation, plants were placed in a mist chamber at 20 to 22°C for 48 hours to facilitate infection, then transferred to a greenhouse maintained at 22 to 28°C to promote disease development. Leaf spot severity was visually evaluated between 7 and 16 days post-inoculation using a 0 to 4 scale: 0 = no symptoms on leaves; 1 = 1–25% leaf area infected; 2 = 26–50% leaf area infected; 3 = 51–75% leaf area infected; and 4 = 76–100% leaf area infected (Mou et al., 2008). In this study, we used the average disease severity as the SLP disease severity index (SDI).

### 2.3 Phenotype data analysis

The experiment was conducted using a randomized complete block design with three replications (pots) per treatment in a greenhouse setting. Disease severity index (DSI) were analyzed using analysis of variance (ANOVA) and a random effects model in META-R v6.0.4, treating genotype as a fixed effect and replication as a random effect. The best linear unbiased estimates (BLUE) were estimated with the model ${\text{Y}}_{ij}=\text{µ}+{\text{Rep}}_{i}+{\text{Gen}}_{j}+\epsilon_{ij}$ , where Y*ij* is the disease response of the j^th^ genotype (Gen*j*) in the i^th^ replication (Rep*i*) and *Eij* is the residual error. The BLUE values were used as phenotype datasets for GWAS analysis. Broad-sense heritability on a genotype-mean basis was calculated using the variance component estimates from the same model, as


$$\text{H\textasciicircum{}}2=\frac{{\text{σ}}^{2}\text{g}}{{\text{σ}}^{2}\text{g }+\frac{{\text{σ}}^{2}e}{n\text{Rep}}}$$

where $\sigma^{2}$ g is the genetic variance and $\sigma^{2}e$ is the prediction error variance, and *n*Rep is the number of replicates. The top 10 accessions were reevaluated for consistency in disease reactions.

### 2.4 Sequencing and SNP calling

Genomic DNA was extracted using the Omega MagBind Plant DNA DS Kit (Omega Bio-tek Inc., Norcross, GA, USA) on a KingFisher Flex automated extraction system (Thermo Fisher Scientific, Waltham, MA, USA). DNA concentration was quantified using a Qubit Fluorometer, and integrity was assessed by 1% agarose gel electrophoresis. Paired-end sequencing libraries were constructed for each spinach accession and sequenced on the Illumina NovaSeq platform at the Beijing Genome Institute (BGI). Whole-genome resequencing (WGR) generated approximately 10 Gb of sequence data per sample, corresponding to ~10× genome coverage. Sequencing reads were aligned to the *Monoe-Viroflay* spinach reference genome (Cai et al., 2021) using the Illumina DRAGEN (Dynamic Read Analysis for GENomics) pipeline (v3.8.4), and SNP calling was subsequently performed. Initial variant filtering was conducted using BCFtools (Li, 2011) with the following parameters: a minimum sequencing depth of 6×, a minimum genotype quality score of 10, and a minor allele frequency (MAF) ≥ 0.05. This resulted in the identification of 4.92 million SNPs across 470 spinach accessions, including 4.88 million SNPs located on the six spinach chromosomes. For downstream analyses in this study, SNP data from 311 spinach accessions with available phenotypic data were extracted and further filtered by removing SNPs with heterozygosity >13.5%, missing data >7.5%, and MAF <1.6%. The final dataset comprised 135,127 high-quality SNPs and was used for genetic diversity and genome-wide association studies (GWAS) (**Supplementary Figure S1**). This dataset has been deposited in the Figshare repository (DOI: 10.6084/m9.figshare.29429405).

### 2.5 Population structure and genetic diversity

Population structure among the spinach accessions was assessed using the model-based clustering method implemented in ADMIXTURE v1.22 (Alexander et al., 2009). Ten-fold cross-validation (–cv=10) was performed for K values ranging from 1 to 10, with 500 bootstrap replications. Based on the lowest cross-validation error and prior studies on similar USDA spinach germplasm (Shi et al., 2017; Bhattarai et al., 2022), two population clusters (K=2) were selected. Accessions with membership probability estimates (Q-values) greater than 0.60 were assigned to a specific population group, while those with Q-values ≤ 0.60 were classified as admixed. Bar plots were generated to visualize population structure.

Genetic diversity and principal component analysis (PCA) were performed using the Genomic Association and Prediction Integrated Tool (GAPIT) version 3 (Wang and Zhang, 2021; https://zzlab.net/GAPIT/index.html). PCA was based on eigenvalue decomposition, with the number of components ranging from 2 to 10. A neighbor-joining (NJ) phylogenetic tree was constructed to assess genetic relationships among accessions. PCA plots were generated using both GAPIT 3 and the ggplot2 package in R.

### 2.6 Association analysis

GWAS were performed using three software platforms and multiple statistical models: (1) Five models implemented in GAPIT version 3, including the generalized linear model (GLM), mixed linear model (MLM), multiple loci mixed model (MLMM), Fixed and Random Model Circulating Probability Unification (FarmCPU), and Bayesian-information and Linkage-disequilibrium Iteratively Nested Keyway (BLINK) (Wang and Zhang, 2021; https://zzlab.net/GAPIT/index.html); (2) Three models—FarmCPU, MLM, and GLM—implemented in rMVP (Yin et al., 2021; https://github.com/xiaolei-lab/rMVP); and (3) Three models—MLM, GLM, and single marker regression (SMR)—implemented in TASSEL version 5 (Bradbury et al., 2007). Significant associations were identified using a Bonferroni-corrected threshold (0.05/total number of SNPs), corresponding to a logarithm of odds (LOD) score of 6.43.

### 2.7 Candidate gene search

Candidate genes were identified by searching within ±50 Kb of each significant SNP based on genome annotations from the *Monoe-Viroflay* spinach reference genome. Genome annotation data were obtained from SpinachBase (http://www.spinachbase.org/) or via FTP access (http://spinachbase.org/ftp/genome/Monoe-Viroflay/).

#### 2.7.1 Genomic prediction

In this study, we performed genomic prediction (GP) under several different scenarios, including: (1) using various randomly selected SNP sets, (2) using GAPIT3 for the entire panel, and (3) using GWAS-derived SNP markers. The GWAS-derived markers were obtained from the entire panel with self-prediction, from GAGBLUP analysis in GAPIT3, and from 75% of the entire panel (Ma et al., 2025).

##### 2.7.1.1 Genomic prediction using different randomly selected SNP sets

Prediction accuracy (PA) for the DSI of SLP was evaluated using seven genomic prediction (GP) models: Bayes A (BA), Bayes B (BB), Bayesian LASSO (BL), Bayesian Ridge Regression (BRR), Ridge Regression Best Linear Unbiased Prediction (rrBLUP), Random Forest (RF), and Support Vector Machine (SVM). All analyses were conducted in the R software environment (Ravelombola et al., 2021; Shi et al., 2021, 2022, 2025). The rrBLUP model was implemented using the rrBLUP R package (Endelman, 2011), while the SVM model was applied using the kernlab R package (Karatzoglou et al., 2004). Bayesian models were run with 5,000 iterations and a 2,000-iteration burn-in period using the BGLR R package (Pérez and De Los Campos, 2014). The RF model was implemented with 100 decision trees using the randomForest R package (Liaw and Wiener, 2002).

Nine randomly selected SNP sets were tested, ranging in size from 4 to 15,000 SNPs, and labeled as r4, r50, r100, r200, r500, r1000, r5000, r10000, and r15000. Each SNP set was evaluated using a five-fold cross-validation scheme, where four folds served as the training population (TP) and one fold as the validation population (VP). Genomic estimated breeding values (GEBVs) were calculated for each of the nine SNP sets across all seven models. Each model–SNP set combination was replicated 100 times. Mean correlation coefficients (r-values) and standard errors (SEs) were computed. Boxplots showing GP model performance across the different SNP sets were generated using the ggplot2 package in R.

##### 2.7.1.2 Genomic prediction using GAPIT3 for the entire panel

The GAPIT3 software package was also used to estimate GEBVs using two models: genomic best linear unbiased prediction (gBLUP) and GWAS-assisted genomic BLUP (GAGBLUP, previously referred to as maBLUP) (Wang and Zhang, 2021; https://zzlab.net/GAPIT/index.html). In this analysis, the entire panel of 311 spinach accessions was used as both the TP and VP to predict GEBVs for DSI of SLP.

##### 2.7.1.3 Genomic prediction using GWAS-derived SNP markers

###### 2.7.1.3.1 GWAS-derived SNP markers from the entire panel

GWAS was first conducted using five models: GLM, MLM, MLMM, FarmCPU, and BLINK. SNP markers significantly associated with DSI were identified from each model using the entire panel of 311 spinach accessions. These GWAS-derived SNPs were then used in GP using a cross-population strategy with 5-fold cross-validation across seven GP models: BA, BB, BL, BRR, rrBLUP, RF and SVM, following the procedure previously described for randomly selected SNP sets.

###### 2.7.1.3.2 GWAS-derived SNP markers using GAGBLUP in GAPIT3

GP was also conducted using the GAGBLUP (BLINK) model in the GAPIT3 package. The entire panel of 311 accessions was divided into two subsets: 75% (233 accessions) as the TP and 25% (78 accessions) as the VP. Phenotypic values of the VP were set to ‘NA’ during model training. Prediction accuracy (r-value) was calculated as the correlation between GEBVs and observed phenotypic values in the VP. This process was repeated five times, and the mean r-value was used to assess model performance. Three prediction scenarios were evaluated: (1) Across-Prediction: SNP markers identified from the training set (233 accessions; average of five GWAS runs) were used to predict the validation set (78 accessions). (2) Cross-Prediction: SNP markers from the training set were used to predict the same training set (233 accessions). (3) Self-Prediction: SNP markers from the entire panel (311 accessions) were used to predict the same set.

###### 2.7.1.3.3 Across- and cross-population prediction using GWAS-derived SNP markers from 75% of the entire panel

The full panel (311 spinach accessions) was again divided into 75% TP (233 accessions) and 25% VP (78 accessions). GWAS was performed on the TP using the BLINK model in GAPIT3. SNP markers with −log_10_(P) > 3.0 were selected for use in GP models. GEBVs were estimated using six models: BA, BB, BL, BRR, RF, and SVM. Both cross- and across-population predictions were performed to predict DSI using the GWAS-derived SNP markers. Each GP model was replicated 100 times per run. The mean correlation coefficient (r-value) between GEBVs and observed phenotypic values was calculated across replications. This procedure was repeated five times, and the mean r-value was used as the final prediction accuracy. Standard errors (SEs) of the r-values were also calculated.

Three prediction scenarios were tested: (1) Across-Prediction: SNP markers identified from the training set (233 accessions) were used to predict the validation set (78 accessions), averaged over five GWAS runs. (2) Self-Prediction: SNP markers from the training set were used in five replications to predict the entire population (311 accessions). (3) Cross-Prediction: SNP markers from the training set were used to predict the same training set. Boxplots depicting the performance of each GP model across SNP sets were generated using the ggplot2 package in R.

## 3 Results

### 3.1 Phenotyping

**Figure 1:**
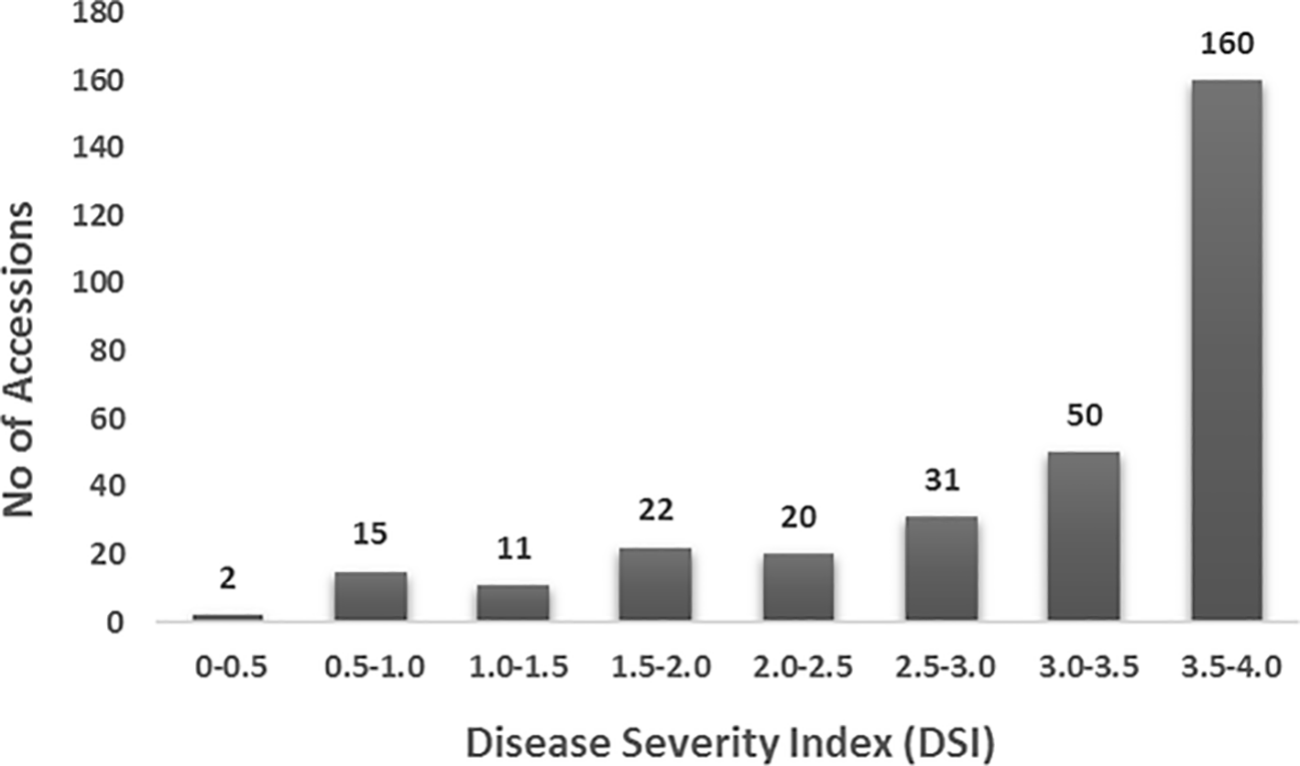
Distribution of disease severity index (DSI) for *Stemphylium* leaf spot in 311 USDA spinach accessions and commercial cultivars inoculated with isolate Sb-1-St001 of *S. vesicarium*. The y-axis represents the number of accessions, while the x-axis shows the DSI on a 0–4 scale.

A total of 311 spinach genotypes, collected from 29 countries, were evaluated for SLP disease under greenhouse conditions. The results revealed a wide range of variation in disease responses (**Figure 1**; **Supplementary Tables 1a, 1b**). The susceptible cultivar ‘Viroflay’ had a disease score of 4.0, while none of the genotypes exhibited complete resistance. Among the USDA accessions and commercial cultivars, diverse disease responses were observed: 36.0% of genotypes were completely susceptible (disease score = 4.0), 31.5% showed high susceptibility (disease score 3.0–3.96), 16.4% moderate susceptibility (disease score 2.0–2.99), 10.6% moderate resistance (disease score 1.0–1.99), and 5.5% high resistance (disease score < 1.0). The overall phenotypic distribution was skewed toward susceptibility (**Figure 1**), indicating that the majority of accessions were susceptible to SLP and highlighting the need to develop new spinach cultivars with SLP resistance for improved spinach production.

The top ten resistant and susceptible genotypes were reevaluated for consistency in disease scoring. The results showed a strong correlation (|r| = 0.68), confirming the reliability of the phenotyping (Liu et al., 2020a). The genotypes with the highest levels of resistance (disease score < 1.0) were: PI 179041, ‘Tasman’, PI 604779, PI 648948, 03_316_Old_7, CPPHIS_3_08 (‘Lazio’), PI 179596, PI 433209, PI 604778, PI 433211, 08_03_316_1_Fay, PI 179597, PI 262161, PI 433207, ‘Silverwhale’, PI 531457, and PI 535897 (**Supplementary Table 1**). Among these, 03_316_Old_7 and 08_03_316_1_Fay are breeding lines developed by the University of Arkansas, while ‘Tasman’ and ‘Silverwhale’ are commercial cultivars. The remaining genotypes are USDA germplasm accessions originating from Belgium, China, France, Hungary, Japan, Poland, Spain, and Turkey. All resistant genotypes were classified within the Q1 or Q1Q2 population structure groups; none of the Q2 group accessions showed high levels of tolerance to *Stemphylium* in this study. ANOVA revealed significant differences among genotypes for disease response (P < 0.001). The broad-sense heritability, calculated on a genotype-mean basis, was high (H² = 0.97), indicating consistent disease scores across replications.

### 3.2 Genetic diversity

**Figure 2:**
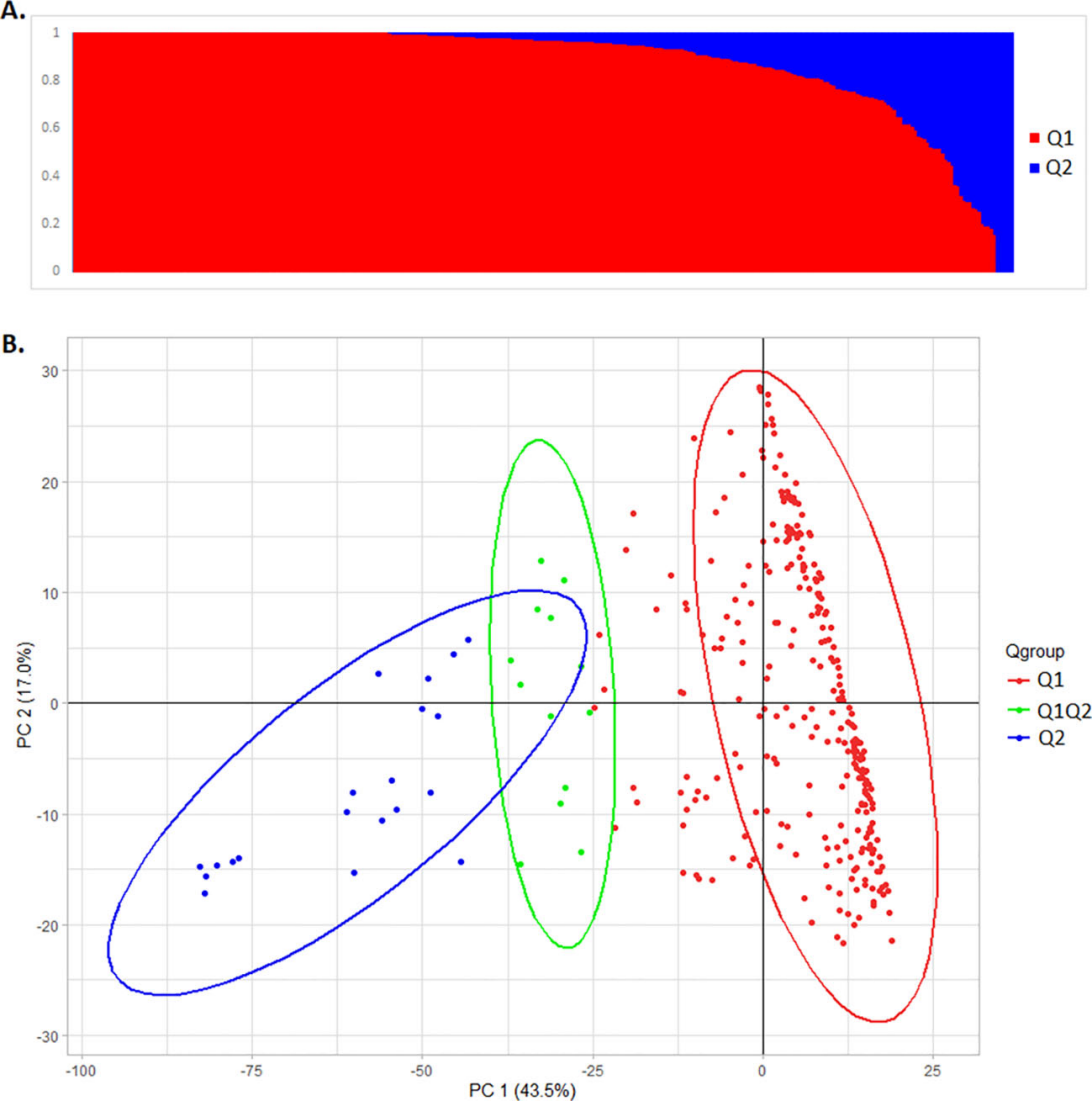
Population structure analysis of 311 spinach GWAS panel evaluated for *Stemphylium* leaf spot resistance. **(A)** The population structure of the GWAS panel separated the worldwide spinach accessions into two major groups: Q1 and Q2 ADMIXTURE v1.22. **(B)** Principal component analysis (PCA) of the spinach GWAS panel shows the first two PC explaining 60.5% of total genetic variation. The accessions were grouped into two major clusters (Q1 and Q2) with some admixed groups (Q1Q2) drawn in R using ggplot2 packages.

Of the 311 spinach accessions analyzed for population structure and genetic diversity using ADMIXTURE v1.22, 278 were assigned to the Q1 cluster, 20 to the Q2 cluster, and 13 were classified as admixed (Q1Q2) (**Supplementary Table S1**; **Figure 2**). The Q2 and Q1Q2 groups included accessions from Asian countries such as India, China, Nepal, Pakistan, and South Korea. One Turkish accession (PI 648938) was assigned to Q2, while all other Turkish accessions clustered in Q1, along with accessions from the United States, various European countries, Iran, Egypt, Syria, Georgia, and Afghanistan. Among the 20 Afghan accessions, one grouped into Q2, one into the admixed Q1Q2 group, and the remaining 18 were placed in the Q1 group. A few accessions from China and India also belonged to the Q2 group. All commercial cultivars, U.S. accessions, and breeding lines from the University of Arkansas were grouped into the Q1 cluster. Principal component analysis (PCA) revealed that the first two principal components accounted for 60.5% of the total genetic variation (PC1 = 43.5%, PC2 = 17.0%), effectively separating the accessions into three groups: Q1, Q2, and Q1Q2 (**Figure 2**). These two PCs were used as covariates in the GWAS model to minimize false positives and false negatives.

Phylogenetic analysis using GAPIT3 also clearly distinguished the three subpopulations. The results were visualized in a 3D PCA plot (**Supplementary Figure S2A**), a PCA eigenvalue plot (**Supplementary Figure S2B**), and both fan-shaped and unrooted phylogenetic trees (**Supplementary Figures S2C, D**). These analyses reinforced the presence of two major subpopulations (Q1 and Q2), as outlined in **Supplementary Table S1**. In the GAPIT3 results, all admixed Q1Q2 accessions were merged into the Q1 group (**Supplementary Figure S3**). A kinship matrix of the 311 accessions and commercial cultivars, also generated using GAPIT3, further confirmed the presence of two distinct genetic groups (**Supplementary Figure S4**). Therefore, a Q-matrix based on the two main subpopulations (Q1 and Q2) was used for the genome-wide association study (GWAS).

### 3.3 Association analysis

In this study, 18 SNP markers associated with the disease severity index (DSI) for *Stemphylium* leaf spot (SLP) resistance in 311 spinach accessions were identified using multiple GWAS models. These included BLINK, FarmCPU, MLMM, MLM, and GLM in GAPIT3; FarmCPU, MLM, and GLM in rMVP; SMR, GLM, and MLM in TASSEL 5; and a t-test. At least one model for each SNP showed a LOD score >6.43, except for SOVchr1_106735636, which showed LOD scores close to 6.0 in two models (**Supplementary Table S2**; **Supplementary Figure S5**), suggesting the presence of QTLs in these SNP regions.

**Figure 3:**
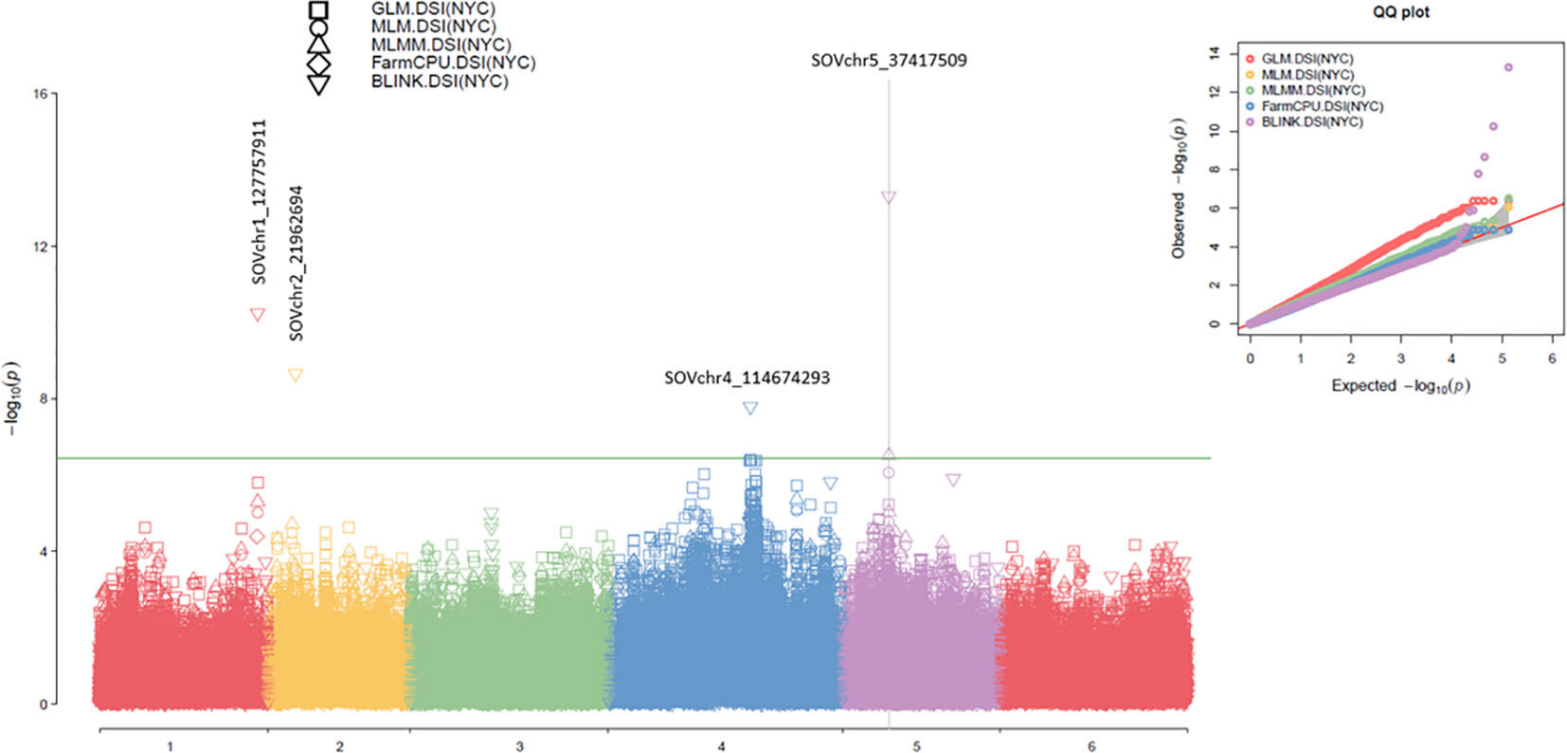
The symphysic Manhattan plot (left) and Q-Q plot (right) compare five GWAS models—GLM, MLM, MLMM, FarmCPU, and BLINK—implemented in GAPIT3 for the disease severity index (DSI) of *Stemphylium* leaf spot resistance in 311 spinach accessions. In the Manhattan plots, the x-axis represents the six spinach chromosomes, and the y-axis shows the LOD scores (−log_10_ P-value). The four most significantly associated SNP markers are also highlighted. In the Q-Q plots, the x-axis indicates the expected LOD scores (−log_10_ P-value), while the y-axis shows the observed LOD scores (−log_10_ P-value).

Among these, four SNPs were selected as the strongest associations for DSI of SLP resistance (**Table 1**; **Figure 3**; **Supplementary Figure S6**). The SNP marker SOVchr1_127757911, located at 127,757,911 bp on chromosome 1, had LOD scores exceeding the Bonferroni-corrected threshold (>6.43) in BLINK (LOD = 10.24) from GAPIT3 and FarmCPU (LOD = 9.49) from rMVP. It also showed significant associations in MLMM (LOD = 5.30), MLM (5.02), and GLM (5.80) in GAPIT3, and GLM (5.33) in rMVP. This SNP explained 10.08% of the phenotypic variance (PVE), indicating that SOVchr1_127757911 is strongly associated with DSI and likely marks a QTL region on chromosome 1.

**Table 1:** List of four SNP markers associated with the disease severity index (DSI) of Stemphylium leaf spot resistance in 311 spinach accessions, identified using multiple GWAS models, including BLINK, FarmCPU, MLMM, MLM, and GLM in GAPIT3; FarmCPU, MLM, and GLM in rMVP; and a *t-*test.

SNP	Chr	Pos	MAF %	LOD[-log(*P*-value)] in GAPIT3					LOD[-log(*P*-value)] in rMVP			LOD[-log(*P*- value)] in Tassel 5			R-square in Tassel 5			PVE (%) (Model)	LOD	Resis. allele	Sus. allele
				BLINK	FarmCPU	MLMM	MLM	GLM	FarmCPU	MLM	GLM	SMR	GLM	MLM	SMR	GLM	MLM		*t*-test		
SOVchr1_127757911	1	127757911	6.75	10.24	4.39	5.30	5.02	5.80	9.49	4.89	5.33	3.59	4.34	4.12	6.03	6.77	7.25	10.08 (blink)	2.61	C	T
SOVchr2_21962694	2	21962694	6.91	8.66	2.32	2.56	2.50	3.18	5.05	2.82	2.74	2.92	1.71	1.85	5.03	3.00	3.49	9.91 (blink)	2.90	T	C
SOVchr4_114674293	4	114674293	4.02	7.79	4.89	5.09	4.82	6.39	2.06	4.03	6.31	6.51	5.86	3.37	9.27	7.97	5.15	33.53 (blink)	4.42	G	A
SOVchr5_37417509	5	37417509	7.23	13.31	3.92	6.51	6.06	5.22	12.15	5.75	8.85	7.94	7.52	4.67	12.15	11.00	8.17	27.12 (blink) 42.7 (mlmm) 55.38 (mlm)	6.74	C	T

The SNP SOVchr2_21962694, located at 21,962,694 bp on chromosome 2, had a LOD score of 8.66 in BLINK and >2.50 in all other models. It explained 9.91% of the phenotypic variance, suggesting a weaker association with DSI and the possible presence of a minor-effect QTL in this region.

The SNP SOVchr4_114674293, located at 114,674,293 bp on chromosome 4, had LOD scores of 7.79 in BLINK and 6.51 in SMR, exceeding the Bonferroni thresholds of 6.43 of LOD. It also showed LOD scores of 6.39 (GAPIT3 GLM), 6.31 (rMVP GLM), and 5.86 (TASSEL GLM), and LOD >4.0 in all nine models except for FarmCPU (2.06) in rMVP and MLM (3.37) in TASSEL. This SNP explained 33.53% of phenotypic variance in BLINK, indicating a strong association with DSI and the presence of a major QTL on chromosome 4.

The SNP SOVchr5_37417509, located at 37,417,509 bp on chromosome 5, showed LOD scores of 13.31 in BLINK and 6.51 in MLMM (GAPIT3); 7.15 in FarmCPU and 8.85 in GLM (rMVP); and 7.94 in SMR and 7.52 in GLM (TASSEL). It exceeded the significance threshold of 6.43 in most models and had LOD >5.2 in all nine models except for FarmCPU (3.92) in rMVP and MLM (4.67) in TASSEL. It explained 27.12% (BLINK), 42.7% (MLMM), and 55.38% (MLM) of the phenotypic variance, indicating a very strong association with DSI of SLP resistance and the presence of a major QTL in this SNP region on chromosome 5.

The *t*-test revealed significant LOD values of 2.61, 2.90, 4.42, and 6.74 for the four SNP markers, respectively (**Table 1**), indicating a significant association between these markers and SLP resistance. Allele distributions of the four SNPs significantly associated with the DSI of SLP resistance across 311 spinach accessions are presented in **Supplementary Figure S7**. For each SNP, the allele associated with lower DSI values (i.e., the resistance allele) was found in fewer accessions, suggesting a strong correlation between the presence of the resistance allele and resistance to *Stemphylium* leaf spot.

#### 3.3.1 Candidate gene search

A total of 61 genes were identified within ±50 kb of the 18 SNPs associated with DSI for SLP resistance (**Supplementary Table S3**). Seven genes located closest to the four key SNPs (SOVchr1_127757911, SOVchr2_21962694, SOVchr4_114674293, and SOVchr5_37417509) are listed in **Table 2**.

**Table 2:** List of seven genes located within 50 kb upstream or downstream and dosest to the four SNP markers identified in **Table 1**, which are associated with the disease severity index (DSI) for Stemphylium leaf spot resistance in 311 spinach accessions.

Gene	Chr	Start_pos	End_pos	Gene name	SNP	Chr	Pos	Distance from gene_start	from gene_end	Comment
SOV1g040270	1	127752039	127753611	CASP-like protein	SOVchr1_127757911	1	127757911	5872	4300	<5kb
SOV1g040280	1	127753403	127755510	photosynthetic NDH subunit of lumenal location 2, chloroplastic				4508	2401	<2.5kb
SOV2g005490	2	21945470	21946359	Unknown protein	SOVchr2_21962694	2	21962694	17224	16335	<17kb
SOV2g005500	2	22027716	22032032	CRS2-associated factor 1, chloroplastic				-65022	-69338	<66kb
SOV4g030080	4	114632566	114633635	Gamma-interferon-inducible lysosomal thiol reductase	SOVchr4_114674293	4	114674293	41727	40658	<41kb
SOV4g030090	4	114718128	114723113	Basic-leucine zipper domain				-43835	-48820	<44kb
no gene in the region of 100 Kb					SOVchr5_37417509	5	37417509			
SOV5g021190	5	37515001	37517838	Fe2OG dioxygenase domain-containing protein				-97492	-100329	<100kb

For SOVchr1_127757911, the nearest genes are SOV1g040270 (Casparian strip membrane domain-like protein, CASP) and SOV1g040280 (photosynthetic NDH subunit of lumenal location 2, chloroplastic), both within ~5 kb of the SNP. CASP-like proteins may be upregulated in response to pathogens or abiotic stress (Apostolova, 2023), suggesting that *SOV1g040270* may be involved in SLP resistance. In contrast, *SOV1g040280* is chloroplast-related and less likely to be involved in disease resistance. For SOVchr2_21962694, the closest gene is SOV2g005490, which has an unknown function. Another nearby gene, SOV2g005500, located ~65 kb away, encodes CRS2-associated factor 1 (chloroplastic), and is unlikely to be associated with SLP resistance. For SOVchr4_114674293, two genes are located within 50 kb: SOV4g030080 (Gamma-interferon-inducible lysosomal thiol reductase) and SOV4g030090 (Basic-leucine zipper domain). Basic-leucine zipper (bZIP) transcription factors have been associated with plant immunity (Noman et al., 2017). Zhang et al. (2021) reported that the bZIP transcription factor *GmbZIP15* facilitates resistance to *Sclerotinia sclerotiorum* and *Phytophthora sojae* in soybean. These findings suggest that SOVchr4_114674293 may be linked to SLP resistance in spinach. No gene was found within 50 kb of SOVchr5_37417509, but the nearest gene, SOV5g021190 (Fe2OG dioxygenase domain-containing protein), is located approximately 97 kb away. Wang et al. (2024) reported that 2-oxoglutarate (2OG)-dependent oxygenases (2OGDs) play important roles in plant disease resistance. Further research is required to determine the functional relevance of this SNP region in *Stemphylium* resistance.

#### 3.3.2 Genomic selection of *Stemphylium* resistance in spinach

##### 3.3.2.1 Genomic prediction using randomly selected SNP sets

**Figure 4:**
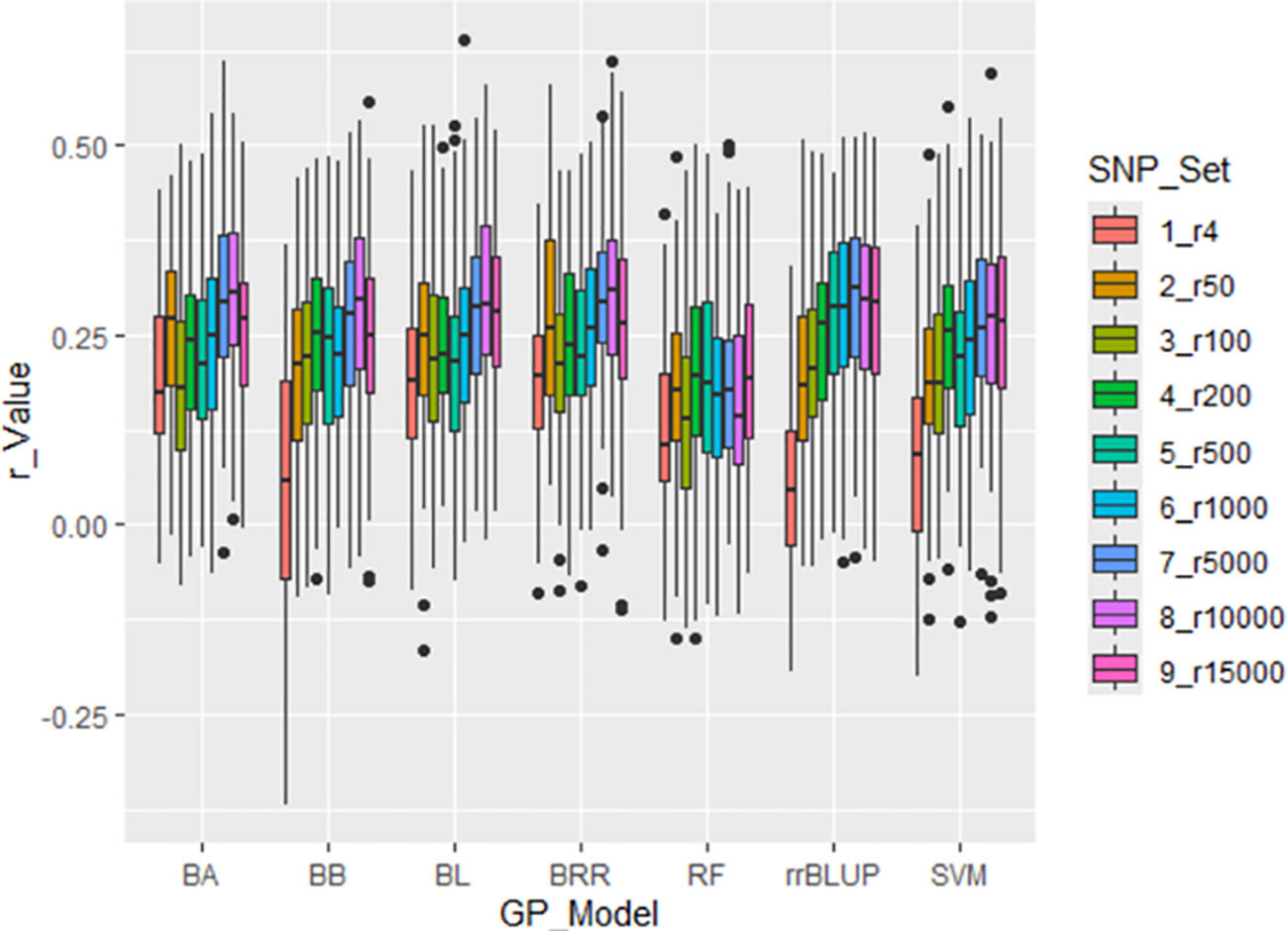
Prediction accuracy (r-value) for *Stemphylium* leaf spot resistance (DSI) in 311 spinach accessions using nine SNP sets (r4 to r15000). Genomic prediction was performed with 5-fold cross-validation, correlating predicted GEBVs with observed phenotypes. Seven models were tested: BA, BB, BL, BRR, rrBLUP, RF, and SVM.

Prediction accuracy (PA), measured as the correlation coefficient (r-value), for the disease severity index (DSI) of *Stemphylium* leaf spot (SLP) resistance was evaluated using nine randomly selected SNP sets, ranging from 4 to 15,000 SNPs (denoted r4 to r15000). Genomic prediction (GP) was performed using a cross-population strategy with 5-fold cross-validation across seven GP models: BA, BB, BL, BRR, rrBLUP, RF and SVM (**Supplementary Table S4**; **Figure 4**).

Across all models, r-values generally increased with the number of SNPs used. However, the average r-value was only 0.12 when using four randomly selected SNPs (r4), and remained below 0.31 even when 1,000 or more SNPs were used—up to 15,000 SNPs—indicating overall low PA for GP of SLP resistance using random SNP sets (**Supplementary Table S4**; **Figure 4**). Among the models, RF consistently showed the lowest prediction accuracy, while rrBLUP exhibited the highest average r-value across the nine SNP sets. These results suggest that genomic selection (GS) for SLP resistance using randomly selected SNP sets is not highly effective.

##### 3.3.2.1 Genomic prediction using GAPIT3 for the entire panel

GP was also performed using the GAPIT3 software package with three models: compressed BLUP (cBLUP), genomic BLUP (gBLUP), and GWAS-assisted genomic BLUP (GAGBLUP, also referred to as aBLUP), using 135,127 SNPs. The full panel of 311 spinach accessions was used as both the training and validation population.

**Figure 5:**
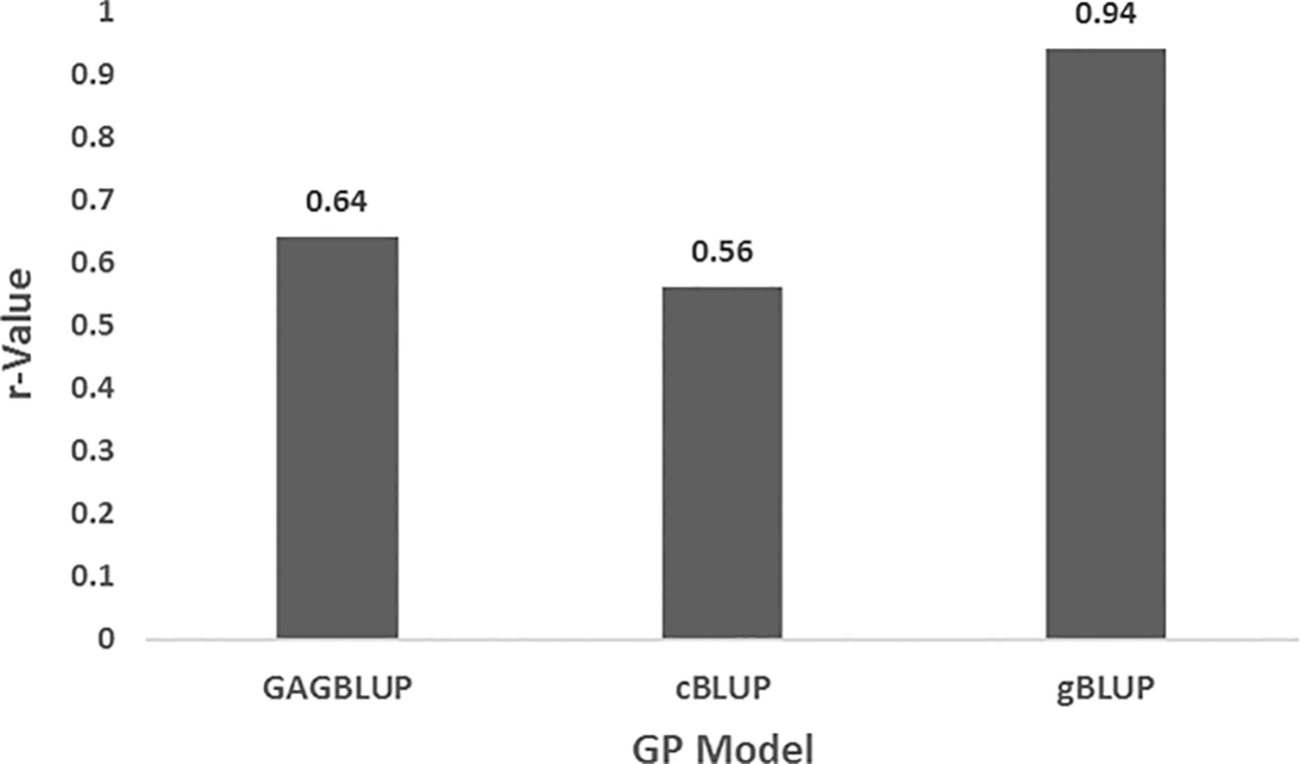
Prediction accuracy (r-value) for *Stemphylium* leaf spot resistance (DSI) in 311 spinach accessions using three GP models—GAGBLUP, cBLUP, and gBLUP—implemented in GAPIT3. The full panel was used as both training and validation set. Accuracy is shown as the correlation between GEBVs and observed phenotypes.

The GAGBLUP (aBLUP), cBLUP, and gBLUP models yielded r-values of 0.64, 0.56, and 0.94 for DSI, respectively (**Figure 5**), indicating high prediction accuracy. These results demonstrate the potential of GS to effectively identify spinach accessions with high levels of resistance to SLP in breeding programs.

##### 3.3.2.1 Genomic prediction using GWAS-derived SNP markers

###### 3.3.2.1.1 GWAS-derived SNP markers from the entire panel (self-prediction)

**Figure 6:**
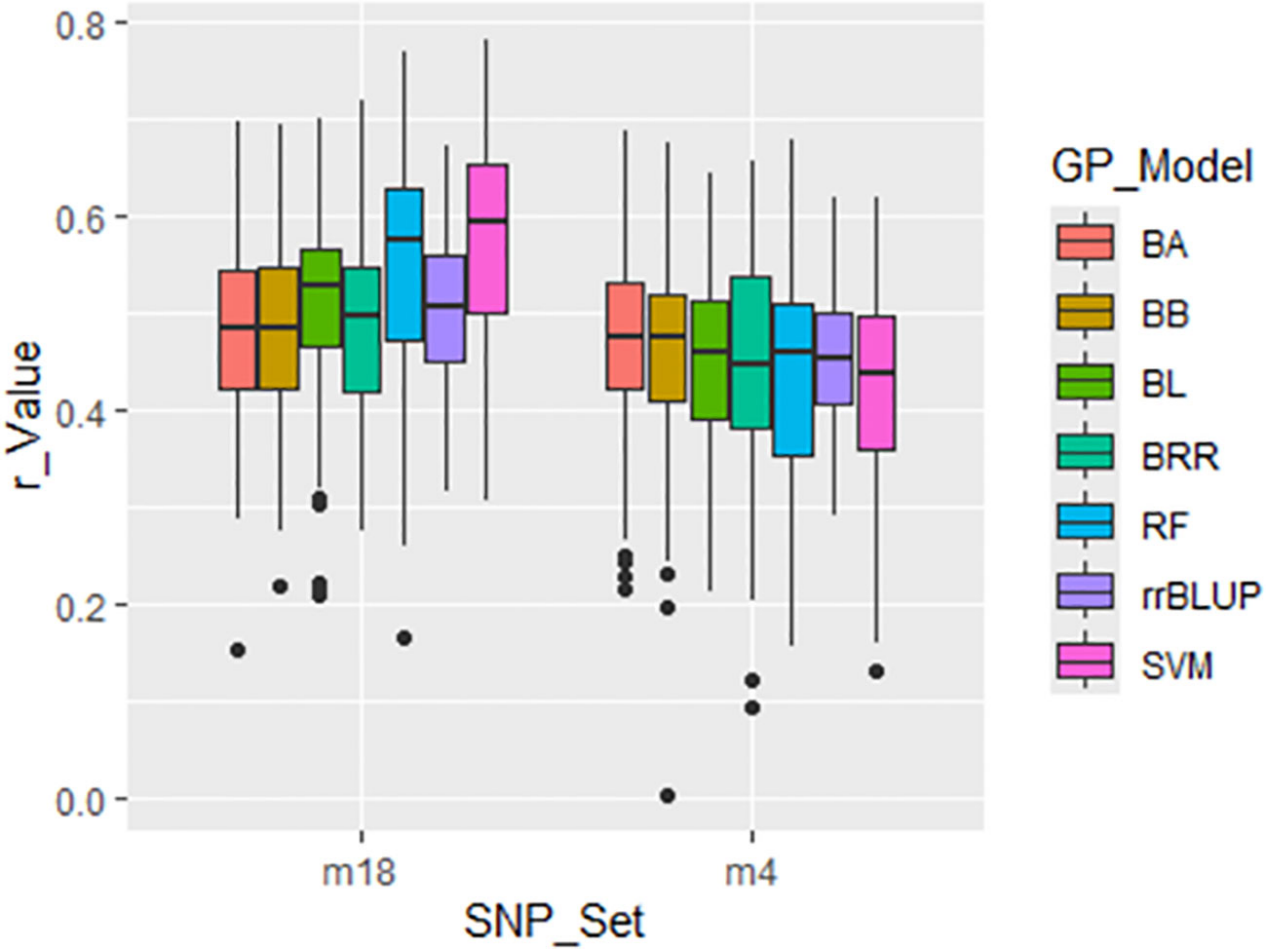
Prediction accuracy (r-value) for *Stemphylium* leaf spot resistance (DSI) in 311 spinach accessions using two GWAS-derived SNP sets: 18 SNPs (m18) and 4 SNPs (m4). A 5-fold cross-validation scheme was used, with accuracy expressed as the correlation between GEBVs and observed phenotypes across seven GP models: BA, BB, BL, BRR, rrBLUP, RF, and SVM).

GWAS was conducted on the entire panel of 311 spinach accessions to identify SNPs significantly associated with DSI of SLP resistance. Two GWAS-derived SNP sets—m4 (4 SNPs) and m18 (18 SNPs)—were evaluated. The GP was performed using a cross-population strategy with 5-fold cross-validation across seven GP models: BA, BB, BL, BRR, rrBLUP, RF and SVM. These sets produced progressively higher prediction accuracies, with r-values of 0.45 and 0.51, respectively, averaged across seven GP models (**Figure 6**; **Supplementary Table S4**). These increasing r-values confirm the relevance of these SNPs to SLP resistance and their potential utility for marker-assisted selection (MAS) and GS.

Since both SNP discovery and validation were conducted within the same population, elevated r-values were expected. However, prediction accuracy is likely to decline in across-population prediction scenarios due to reduced linkage disequilibrium and differing population structures. Subsequent sections assess the performance of these GWAS-derived SNP markers in cross- and across-population predictions.

###### 3.3.2.1.2 GWAS-derived SNP markers using GAGBLUP in GAPIT3

**Figure 7:**
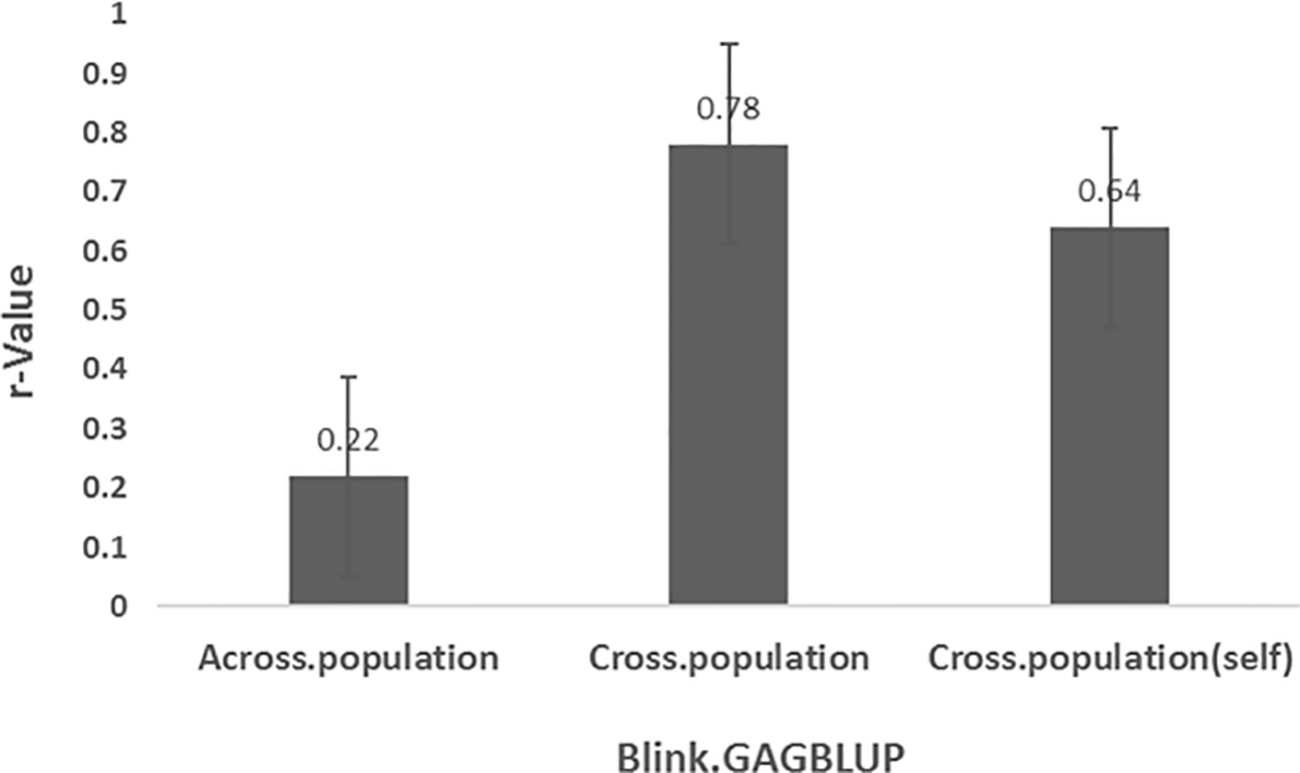
Prediction accuracy (r-value) for *Stemphylium* leaf spot resistance (DSI) in 311 spinach accessions using the GAGBLUP model (equivalent to MaBLUP or BLINK) in GAPIT3. Three scenarios are shown: (1) Across-prediction—markers from 75% of accessions (n=233, averaged over five GWAS runs) used to predict the remaining 25% (n=78); (2) Cross-prediction—same 75% used to predict themselves; (3) Cross-population (self)—full set (n=311) used for both training and predictin.

Genomic prediction was performed using the GAGBLUP model (also referred to as MaBLUP or BLINK) in GAPIT3 (**Figure 7**). Three prediction scenarios were evaluated:

- Across-population prediction: r = 0.22
- Cross-population prediction: r = 0.78
- Cross-population (self): r = 0.64

The substantially lower r-value (0.22) in the across-population prediction indicates reduced prediction accuracy when SNP markers identified in one population are applied to an independent validation set. Nevertheless, the high r-values in the other two scenarios confirm that these SNP markers are indeed associated with SLP resistance and can be effective under within-population GP frameworks.

###### 3.3.2.1.3 Across- and cross-population prediction using GWAS-derived SNP markers from 75% of the Panel

To further evaluate prediction robustness, GWAS was performed using 75% of the panel (233 accessions) as the training set, and the identified SNPs were used to predict DSI of SLP resistance under three scenarios:

1. Across-population prediction: SNPs from the 75% training set were used to predict the remaining 25% (78 accessions).
2. Cross-population prediction: SNPs were used to predict the training population itself.
3. All (self)-prediction: SNPs from the 75% training set were used in five replications to predict the full set of 311 accessions.

**Figure 8:**
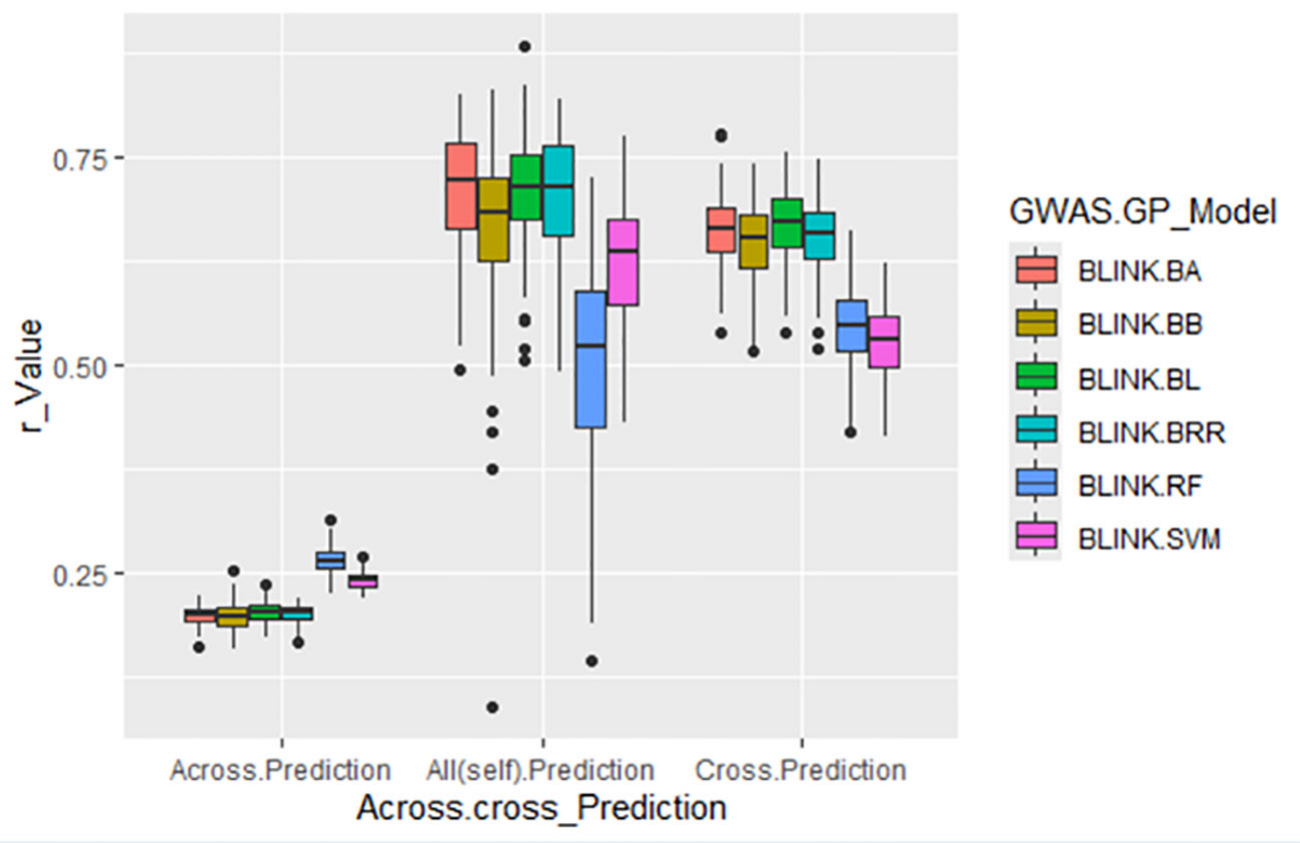
Prediction accuracy (r-value) for *Stemphylium* leaf spot resistance (DSI) in 311 spinach accessions using GWAS-derived SNP markers under three scenarios: (1) Across-prediction—markers from 75% of accessions (n=233, averaged over five GWAS runs) used to predict the remaining 25% (n=78); (2) All (self)-prediction—markers from the same 75% used to predict the full set (n=311); (3) Cross-prediction—markers from the 75% training set used to predict themselves.

The cross-population prediction scenarios (Cross-Prediction and All(Self)-Prediction) yielded consistent r-values >0.60, with averages of 0.62 and 0.65, respectively, across six GP models (**Supplementary Table S5**; **Figure 8**). These results support the association between the selected GWAS-derived SNPs and DSI resistance, demonstrating the usefulness of incorporating GWAS-informed markers into GS strategies for enhancing SLP resistance in spinach. In contrast, across-population prediction yielded a low average r-value of 0.22 across six models, highlighting the limitations of transferring GWAS-derived SNP markers across distinct genetic backgrounds for GS of SLP resistance.

## 4 Discussion

In recent decades, leaf spot diseases have become a significant economic concern in spinach production, as well as in several other crops. With resistance to *Stemphylium* leaf spot (SLP) emerging as a priority in spinach breeding programs, this study evaluated a large set of USDA spinach germplasm, commercial cultivars, and breeding lines under greenhouse conditions to identify highly tolerant accessions and discover DNA markers associated with resistance. Screening for resistance across this panel revealed several highly tolerant accessions and wide variation in disease responses. Accessions such as PI 179041, Tasman, PI 604779, PI 648948, 03_316_Old_7, CPPHIS_3_08 (Lazio), PI 179596, PI 433209, PI 604778, PI 433211, 08_03_316_1_Fay, PI 179597, PI 262161, PI 433207, Silverwhale, PI 531457, and PI 535897 showed low disease ratings (below 1.0) (**Supplementary Table S1**). These highly resistant sources are valuable for incorporating *Stemphylium* resistance into spinach breeding programs and for further investigation into the genetic and molecular mechanisms of resistance. However, most of the tolerant accessions identified in this study did not match earlier reports, which were based on different isolates and environments (Mou et al., 2008; Wadlington et al., 2018). Therefore, further evaluations under multiple environments and field trials are warranted.

The broad-sense heritability, calculated on a genotype-mean basis, was high (H² = 0.97), suggesting that a large portion of the phenotypic variation in disease response is genetically controlled and amenable to improvement through breeding and selection. Large variations and lack of stability in phenotypic responses are common in plant disease screening, especially in field evaluations (Poland and Rutkoski, 2016; Bhattarai et al., 2022), due to genotype × environment (G×E) interactions and the complex interplay of pathogen populations and environmental factors. This study evaluated the GWAS panel using single pathogen isolates under controlled greenhouse conditions, providing a more homogeneous environment and consistent responses between replicates, thus resulting in higher heritability estimates. Nevertheless, these estimates should be cautiously interpreted, as the screening was conducted in a single greenhouse and may not capture environmental variability.

Organic spinach production accounts for approximately half of the total spinach production in the United States and urgently requires the development of cultivars resistant to *Stemphylium* for sustainable production. Previous screening trials also reported broad genetic variation in resistance to SLP (Mou et al., 2008). A subsequent GWAS study identified a few SNPs associated with resistance (Shi et al., 2016). However, earlier studies did not utilize an assembled and annotated spinach genome. This study, by contrast, used the latest reference genome (Cai et al., 2021), although it did not compare identified regions with earlier reports due to inconsistencies in resistance responses among accessions across studies. To date, few studies have reported SNP markers associated with *Stemphylium* resistance in spinach. Identifying and validating SNPs linked to resistance will provide useful molecular tools for selection and introgression of resistance loci.

Different genomic selection (GS) models vary in their assumptions regarding marker effects, so prediction accuracy (PA) depends on the phenotype and underlying genetic architecture (Daetwyler et al., 2010; Habier et al., 2013). Therefore, evaluating various marker sets and models helps determine the most effective strategy for a given trait. In this study, Bayesian models showed higher PA for both 4- and 18-SNP GWAS-derived marker sets (**Figure 6**; **Supplementary Table S4**), which is consistent with their advantage in predicting traits governed by a few major QTLs (Daetwyler et al., 2010). The rrBLUP model, which assumes equal variance across markers and accounts for relatedness, showed lower PA for some traits, including field resistance to downy mildew in spinach (Shikha et al., 2017; Islam et al., 2020; Bhattarai et al., 2022).

Interestingly, small GWAS-derived SNP sets (4 and 18 SNPs) outperformed the full SNP set in prediction accuracy (**Figure 6**; **Supplementary Table S4**), suggesting that a smaller, targeted marker set can be more effective and cost-efficient. The higher PA observed with these small sets is likely due to reduced overfitting, a phenomenon also reported for downy mildew and white rust resistance in spinach (Bhattarai et al., 2022; Shi et al., 2022) and for stripe rust resistance in wheat (Merrick et al., 2021). These results highlight the practical value of using optimized SNP sets for GS to predict resistance to SLP at a lower genotyping cost.

Three types of GWAS-derived SNP markers were analyzed in this study: (1) GWAS-derived SNP markers from the entire panel, (2) GWAS-derived SNP markers identified using the GAGBLUP model in GAPIT3, and (3) GWAS-derived SNP markers from 75% of the panel (Ma et al., 2025) (**Supplementary Tables S4**, **S6**; **Figures 4**–**8**). In the first scenario, the full panel of 311 spinach accessions was used to identify SNPs significantly associated with SLP disease severity index (DSI), and the same panel was used in a five-fold cross-validation framework across seven GP models: BayesA (BA), BayesB (BB), BayesLASSO (BL), Bayesian Ridge Regression (BRR), rrBLUP, Random Forest (RF), and Support Vector Machine (SVM). Two GWAS-derived SNP sets—m4 (4 SNPs) and m18 (18 SNPs)—were selected for genomic prediction. Both sets yielded progressively higher prediction accuracies, with r-values of 0.45 and 0.51, respectively (**Figure 6**; **Supplementary Table S4**), averaged across the seven models, confirming their association with SLP resistance and potential for MAS and GS.

In the second and third scenarios, either GAGBLUP or the six non-linear GP models produced high cross-population prediction accuracies (r-values: 0.62–0.78) (**Figure 7**). However, when these SNP sets were tested across distinct populations (i.e., cross-population GS), the average r-value dropped to 0.22 (**Figure 8**), indicating limited transferability of GWAS-derived markers across genetically diverse panels (**Supplementary Table S5**). This suggests that population structure and genetic background must be considered when applying GS for SLP resistance.

## Data Availability

The data generated in this study are provided in the main tables, figures, and supplementary files. Whole-genome resequencing (WGR) data aligned to the reference genome are available at NCBI under BioProject ID: PRJNA860974. SNP data, generated using the Monoe-Viroflay spinach reference genome (Cai et al., 2021), are available in the Figshare repository at https://doi.org/10.6084/m9.figshare.29429405.v1. Accession numbers used in this study are listed in both the main text and **Supplementary Materials**.
